# Increased user engagement on YouTube for loot box content and its potential relevance for behavioural addictions

**DOI:** 10.1038/s41598-025-01482-5

**Published:** 2025-05-15

**Authors:** Elke Smith, Yannik Poth, Kara S. Z. Sohns, Kai Kaspar, Jan Peters

**Affiliations:** 1 Department of Psychology, Biological Psychology, University of Cologne, Cologne, Germany; 2 Department of Psychology, Social and Media Psychology, University of Cologne, Cologne, Germany

**Keywords:** Loot box, User engagement, YouTube, Gaming, Gambling-like mechanisms, Gambling, Human behaviour, Psychology, Risk factors

## Abstract

Video games frequently contain loot boxes, i.e. virtual in-game items sharing structural similarities with gambling. On YouTube^©^, there are multi-million subscriber channels prominently featuring loot box-related content. A gamblification of digital games may increase player engagement, and we tested if user engagement on YouTube is linked to loot box content. We extracted aggregate user engagement measures from more than 22 thousand YouTube gaming videos with and without focused display of loot boxes. Principal component analysis was used to reduce dimensionality and derive components reflecting overall and sustained, and relative user engagement, respectively. Confirming our pre-registered hypothesis (see https://osf.io/nh7zr), a significant effect of loot box content on the first principal component was found, reflecting higher overall and sustained user engagement for videos featuring loot box content. This increased engagement may be linked to the gambling-like properties of the reward structure conveyed by loot boxes. Publicly available user data may serve as an early indicator of potential changes in problematic internet use and gambling-related behaviour.

**Supplementary Information:**

The online version contains supplementary material available at 10.1038/s41598-025-01482-5.

## Introduction

During the last decade, developers of many video games have implemented a new method to generate revenue in video games. So-called *loot boxes* are digital containers that contain virtual items that are useful to the player. In most video games, loot boxes can be acquired in three (not mutually exclusive) ways: directly using real money, indirectly by purchasing in-game currency, which is then used to buy loot boxes, and through in-game currency earned by playing and completing tasks within the game. The content of loot boxes is not predictable and unknown to the player at the time of purchase^1,2^. Loot boxes offer players a way to obtain specific game items, such as weapons, special abilities, or cosmetic objects. While cosmetic items usually only serve as a status symbol or expression of one’s identity within the video game, functional items such as weapons have a direct influence on the gameplay and can improve a player’s position in the game or enable faster progress^3^.

Loot boxes represent a special form of in-game purchases, and can be distinguished from *direct sales*, in which a specific item is purchased directly. In-game purchases are also referred to as microtransactions, describing the seemingly insignificant purchase amount of such options^4^. In-game purchases constitute an important source of revenue for game developers in the face of stagnating retail prices in the video game segment and increasingly expensive productions^5^. While for some video games, loot boxes generate additional post-sales revenue, many free-to-play video games generate revenue entirely from loot boxes and other microtransactions. In 2020, more than 50% of the top games in the Google Play Store and Apple’s App Store games contained loot boxes^2^. The implementation of loot boxes seems to be especially profitable for developers of mobile games since adolescents exposed to loot boxes spend more on mobile video games^6^. Revenue from loot boxes is not evenly distributed among users. Around 5% of buyers generate more than half of the revenue^7^. Some authors suggest that these high-consumption gamers, also known as “whales”, are primarily pathological gamblers, pathological video gamers, or other at-risk groups^8^. Individuals who gamble appear to spend more on loot boxes compared to individuals who don’t^9^. The appeal and utility of loot boxes, especially those containing rare or legendary items, depend on a player’s opportunity to use the items in meaningful ways. This dynamic may create a feedback loop, where acquiring powerful items encourages prolonged gameplay to maximise an item’s potential. The value that players attribute to loot box content, driven by perceived higher power and in-game achievement and progression may reinforce involvement in the game and are likely key motivators for loot box purchases.

Loot boxes are discussed as having gambling-like properties, since they share structural similarities with games of chance, such as randomness, unpredictability of the outcome, and hope for a valuable prize^10–13^. As in classical gambling formats, the chance-based reward structure of loot boxes represents a form of intermittent reinforcement^11^, which is thought to be a primary driver of the addictive potential of gambling^14^. The specific implementation of loot boxes in video games is also often reminiscent of classic gambling environments, such that rewards and reward anticipation are accompanied by exciting sounds, visual effects, and other dramatisations during opening^3^. Obtaining items from loot boxes triggers reward-related responses and arousal comparable to those elicited by slot machines^15^.

While many manufacturers of video games claim that loot boxes are harmless items with no real value^16,17^, studies demonstrate links between loot-box purchasing behaviour and gaming addiction, with meta-analyses reporting correlations in the small to medium range^7,18^. Further, time and money spent on loot boxes are correlated with problematic gambling behaviour and maladaptive gambling-related beliefs^19^. The use of loot boxes appears to influence the frequency of video gaming and gambling, and loot boxes pose a risk of developing symptoms of problem gambling by promoting greater involvement in video games^20^. A recent study reports loot box use to be a strong predictor of initiating gambling behaviour^9^.

On video platforms such as YouTube, gaming channels often publish so-called gameplay videos, i.e. recordings of players playing video games and commenting on the gameplay as they play. Platforms such as YouTube offer consumers ways to engage with and respond to content. For instance, it is possible to rate a video with a *like*, or to comment on a video. The Consumers’ Online Brand-Related Activities (COBRA) model provides a framework for describing user engagement, differentiating between the three levels of consumption, contribution and creation, with consumption as minimal level of engagement (e.g. viewing), contribution as medium level (e.g. liking, commenting), and creation as highest engagement level (e.g. publishing content)^21,22^.

In the last few years, the trend to create videos that focus on loot box openings has emerged. YouTube is one of the most visited websites^23^, and there are multi-million subscriber video streams focusing on both gameplay content and on the opening of loot boxes. This again resonates with online gambling content, where a similar trend to watch others gamble has emerged^24^. There is an online market for people who enjoy watching others gamble, and some gamblers report that watching others gamble inspires one’s own gambling^25^. With regard to video games, buyers of loot boxes report feelings of excitement during openings^26^, and viewers of gaming videos, possibly identifying themselves with the streamer, might experience some excitement from simply watching loot boxes being opened.

Based on these observations, the present work aimed to assess whether user engagement with gaming videos on YouTube is in part modulated by the display of loot boxes in such videos. (Behavioural) addiction is characterised by repeated and compulsive engagement with rewarding stimuli^27^, frequently accompanied by impulsive responding^28,29^. Assuming that loot boxes resemble gambling, they may trigger addiction-like behaviour, manifesting as stronger and faster engagement with such content. Therefore, we predicted that user engagement would be higher for videos that contain loot boxes compared to videos without, and preregistered this prediction (https://osf.io/nh7zr). To test this hypothesis, we scraped publicly available video statistic data from selected YouTube gaming channels and quantified user engagement for videos with and without loot boxes. We operationalised user engagement via dimensionality reduction (principal component analysis) across a set of various user engagement measures (see “User engagement variables” section), and predicted higher component values for videos with compared to without loot box content (see “Principal components” section).

## Methods

### Procedure

#### Selection of gaming channels

For the analysis, English-speaking YouTube gaming channels were selected that featured video games prominently featuring loot boxes as a significant component of gameplay^11^. The video game titles were gathered through literature reviews and internet searches. In a subsequent step, we collected video statistics data for the videos of the respective channels. Hereto, a fresh instance of the Chromium web browser (version 103.0.5040.1, https://www.chromium.org/) was used to ensure that the browser was cookie-free, thereby preventing any pre-existing user behaviour from influencing the YouTube search results. Further, a Virtual Private Network (VPN) tunnel provided by NordVPN (version 6.45.10.0, https://nordvpn.com/) was employed to obscure the IP address of the device used for the search. Next, channel searches using specific keywords were conducted on the YouTube website. The term “opening” in the context of video games is commonly used to describe the action of opening a loot box, for instance, “pack opening” in the game FIFA or “mega box opening” in the game Brawl Stars. By inputting the game titles along with the keyword “opening” into the YouTube search bar, relevant search results were retrieved. After sorting the search results by relevance, verified English-language channels (i.e. official channels of creators, artists, companies, or public figures) featuring at least five videos showcasing the opening of loot boxes and five gaming videos devoid of loot box video content were considered for the analysis. Of the 17 channels pre-registered for analysis (see https://osf.io/nh7zr), 3 channels were excluded due to insufficient focus on loot box content. These channels did not prominently feature loot box openings and hence were not considered. This resulted in the inclusion of 14 channels (see Table 1).

**Table 1:** Selected YouTube gaming channels.

Channel no.^b^	Main game	Genre	N^a^			%
			Subscriber	Views	Videos	Loot
1	Brawl Stars	Battle arena	912,000	202,126,570	960	7.81
2	Brawl Stars	Battle arena	1,400,000	444,832,418	710	11.83
3	Brawl Stars	Battle arena	453,000	120,029,407	1727	22.76
4	Brawl Stars	Battle arena	2,570,000	668,131,822	732	5.87
5	Counter-Strike	FP shooter	3,110,000	1,040,472,967	578	19.03
6	Counter-Strike	FP shooter	2,110,000	699,151,593	1791	8.71
7	FIFA	Football	3,670,000	524,840,746	206	33.01
8	Forza	Racing	1,370,000	548,491,472	3899	0.41
9	Forza	Racing	4,870,000	2,139,844,721	3223	0.19
10	NBA 2K	Basketball	403,000	125,285,641	2828	30.09
11	NBA 2K	Basketball	3,120,000	1,037,875,186	1162	14.63
12	Overwatch	FP shooter	349,000	126,262,461	836	1.44
13	World of Tanks	Tank MMO	668,000	417,738,767	1752	0.40
14	World of Tanks	Tank MMO	230,000	117,293,702	2293	0.44
Total			25,235,000	8,212,377,473	22,697	

*FP* first-person, *MMO* massively multiplayer online game.

^a^May 2022.

^b^The channels have been assigned numbers for confidentiality. The names of the channels can be provided upon reasonable request.

#### Data collection

The video statistics data were collected by means of web scraping, a technique used to extract data from the internet and store it for later retrieval or analysis^30^. The data were downloaded using Google’s YouTube Data API with a custom Python script (version 3.10.0, https://www.python.org/), and the *Requests* library (version 2.26.0, https://pypi.org/project/requests/). The script collected channel statistics, including the number of channel subscribers, video statistics, including the number of views, likes and comments for videos, and timing of likes for videos of a channel, and comment statistics, including the timing of comments, the number of comment likes, comment replies and reply likes. At the time of data collection (6 days, 5 h and 3 min, from 5th to 20th of May 2022), the channels had an average of 1,802,500 subscribers (*SD* = 1,462,884.39) and 586,598,390.93 video views (*SD* = 548,136,071.00).

#### Data exclusion

Videos that did not contain information regarding comments or likes (presumably due to the deliberate concealment of such content by channel owners) were excluded. Further, we excluded videos for which the fit of the comment latency model was below *R*^2^ ≤ 0.6 (see “Comment latency model” section).

#### Classification of loot box video content

Videos were categorised as containing substantial loot box content versus minimal or no loot box content. If a video did not contain any action of loot box opening or the act of opening a loot box was incidental and not relevant to the video’s content (i.e. the opening of a loot box happened without any focus on it), it was labelled as containing minimal or no loot box content. The classification was done manually, whereby each video was screened using YouTube’s progress bar to determine the presence and significance of loot boxes. Following the data collection procedure as described in “Data collection” section resulted in a total of 22,697 videos, of which 2001 were classified as loot (9%), and 20,696 (91%) as non-loot videos.

### Measures

#### User engagement variables

The primary objective of the study was to examine whether user engagement differed between videos with and without loot box content. User engagement was defined based on various metrics available on YouTube, including video views, likes, comments, comment likes, and comment replies. Since the number of likes, comments and views increases over time and differs between channels, they are not suitable for comparing videos from different time points and channels. Therefore, we calculated two relative measures from the absolute numbers of likes, comments and views per video: the *like frequency* as video likes per 1000 video views, and the *comment frequency* as the number of comments per video in relation to 1000 video views. Further, means of comment likes, comment replies, and reply likes were calculated for each video to provide additional insight into user engagement. The video statistics and the derived user engagement variables for loot and non-loot videos, respectively, are listed in Table 2. Data within each channel were *z*-standardised to facilitate comparisons between channels with varying levels of engagement. We also considered the temporal pattern of user engagement, specifically the frequency of comments over time within the first 7 days after video publication (*comment latencies*). We therefore calculated the interval between the time a video was uploaded and the time a comment was published. We binned the *comment latencies* into 8-h time windows and modelled the temporal evolution of the comment frequencies within the first 21 time bins after video release as exponential decay:

$$y\left( x \right) = y0 - a*e^{{\left( { - k*x} \right)}} + a$$

with intercept *y*0, asymptote *a*, and decay rate *k,* using optimize.curve_fit of the *SciPy* package for *Python* (version 1.9.0, https://scipy.org/). The intercept *y*0 reflects how many comments were posted in the first eight hours following the publishing of a video. The decay rate *k* models drop in the number of comments per eight hours. The asymptote *a* reflects the frequency of comments per 8 h, to which the function converges to after 7 days. Goodness-of-fit was evaluated based on *R*^2^.

**Table 2:** YouTube video statistics (means and standard deviations) for loot and non-loot videos.

	Loot	Non-loot	Difference in %^a^
Views	431,189.48 (942,077.81)	248,866.16 (536,951.45)	53.62
Likes	12,268.65 (23,892.17)	7095.74 (14,226.19)	53.43
Comments	860.32 (1451.25)	647.53 (1164.23)	28.22
Comment likes	2.36 (2.62)	2.80 (2.56)	− 17.05
Comment replies	0.45 (0.32)	0.44 (0.27)	2.23
Reply likes	0.43 (0.37)	0.6 (0.50)	− 33.01
Comment frequency^b^	9.75 (19.50)	5.54 (9.14)	55.07
Like frequency^b^	38.58 (21.47)	34.19 (15.89)	12.07
Latency model y0	11,783.66 (55,482.65)	6955.05 (62,565.52)	51.54
Latency model slope	2.33 (1.69)	1.98 (1.34)	16.24
Latency model asymptote	11,783.66 (55,482.65)	6955.05 (62,565.52)	51.54

^a^Difference for loot minus non-loot videos.

^b^Counts per 1000 views.

#### Principal components

The present data set consisted of many interrelated variables (e.g. a video with more views will tend to have more comments). Principal component analysis (PCA) provides a method to summarise a large set of related variables into a smaller set of uncorrelated principal components, by transforming the data such that the resulting components explain a maximal amount of variance. To reduce redundancy among the user engagement measures, we therefore conducted a PCA, including the variables view count, like count, comment count, comment likes, comment replies, reply likes, comment frequency, like frequency, and the three parameters from the comment latency model (comment latency intercept, comment latency asymptote, and comment latency slope). PCA was conducted using the *scikit-learn* package for *Python* (version 1.1.0, https://scikit-learn.org/)^31^. The number of components for statistical analysis was determined by visual inspection of the scree plot (see Fig. S2 of the Supplementary Material) and identifying the maximum position of the elbow bend.

#### Statistical analysis

To examine the relationship between loot box video content and user engagement while accounting for variability related to specific channels, a linear mixed model (LMM) was computed for each of the two components (see “User engagement variables” section), with loot box video content as fixed factor and channel as random factor. The LMMs were specified as a random-intercepts and random-slopes model for the variable “channel”, to account for baseline differences in user engagement across channels and differences in the magnitude of effects of loot box content across channels. The model was implemented with R (version 4.2.1, https://www.r-project.org/) and the R libraries lme4 (version 1.1-15, https://cran.r-project.org/web/packages/lme4/) and lmerTest (version 3.1-3, https://cran.r-project.org/web/packages/lmerTest/), using Restricted Maximum Likelihood (REML) estimation, with an adjusted significance level of α_adjusted_ = 0.025 (significance level of α = 0.05 with Bonferroni correction). Since the Levene test indicated deviations from homogeneity of variances for the first (*W* = 106.69, *p* = 0.00) and second component (*W* = 25.56, *p* = 0.00), the data were square-root transformed before entering the LMMs. Visual inspection of the data suggested minor deviations from normality after transformation (see Figs. S2 and S3 of the Supplementary Material).

## Results

### Comment latency model

We excluded 410 videos (1.8%) due to poor variance explanation by the comment latency model (*R*^2^ ≤ 0.6, see “Data exclusion” section), leaving 22,287 videos for consideration in further analyses. Goodness-of-fit was good for videos with loot box content (median *R*^2^ = 0.99, *MAD* = 0.006), and without loot box content (median *R*^2^ = 0.99, *MAD* = 0.007). The channels contained between 6 and 827 videos with loot box content, amounting to 1964 videos (9%).

### Intercorrelations

**Fig. 1:**
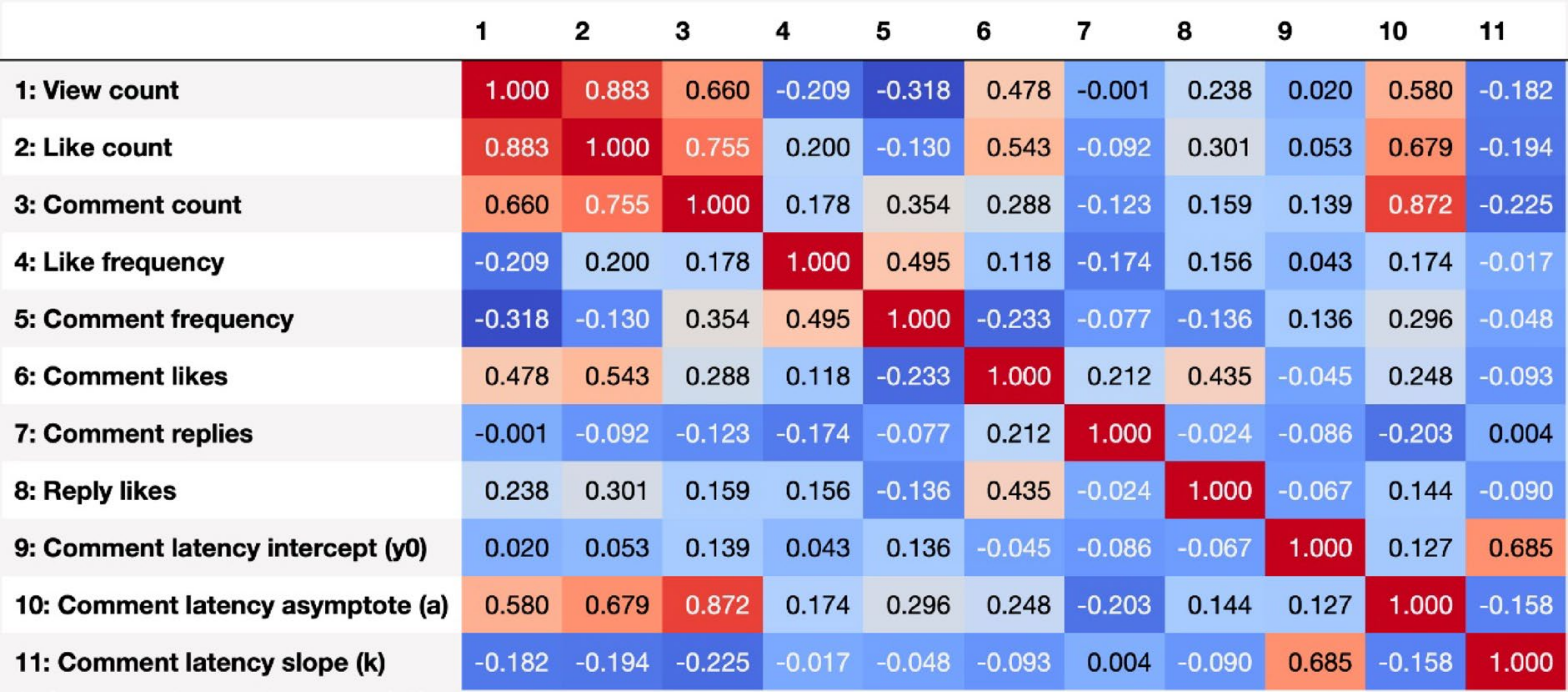
Correlation matrix for the user engagement variables. y0, a and k are parameters of an exponential decay model for the comment latencies.

The full correlation matrix of user engagement measures is provided in Fig. 1. Moderate positive correlations (0.4 ≤ *r* < 0.6) were observed between views and comments, likes and comments, likes and the asymptote of the comment latency model, and between the intercept and decay rate of the comment latency model. Very strong positive correlations (*r* ≥ 0.8) were observed between views and likes, and between comments and the asymptote of the comment latency model.

### Principal component analysis

**Fig. 2:**
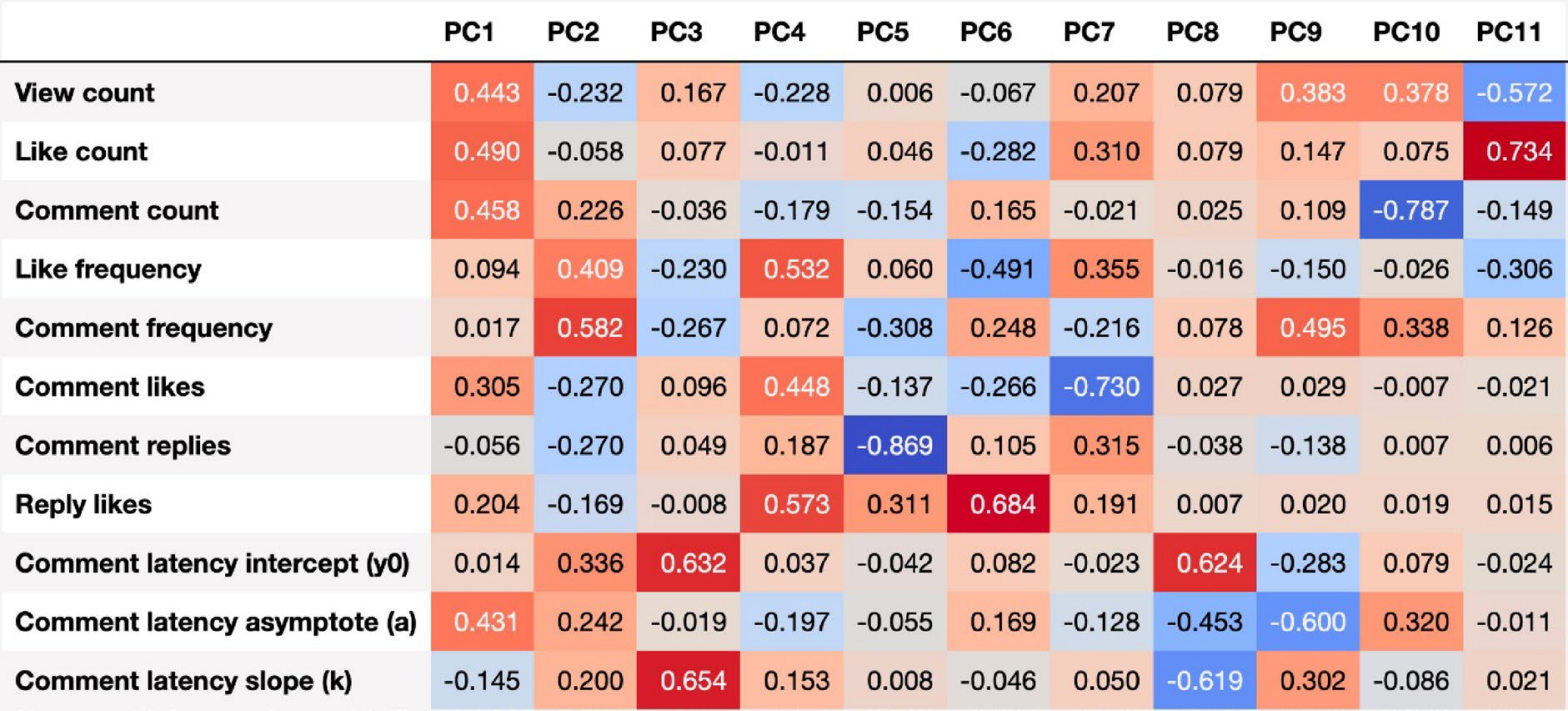
Eigenvector matrix of the user engagement variable loadings onto the principal components (PC). y0, a and k are parameters of an exponential decay model for the comment latencies.

**Fig. 3:**
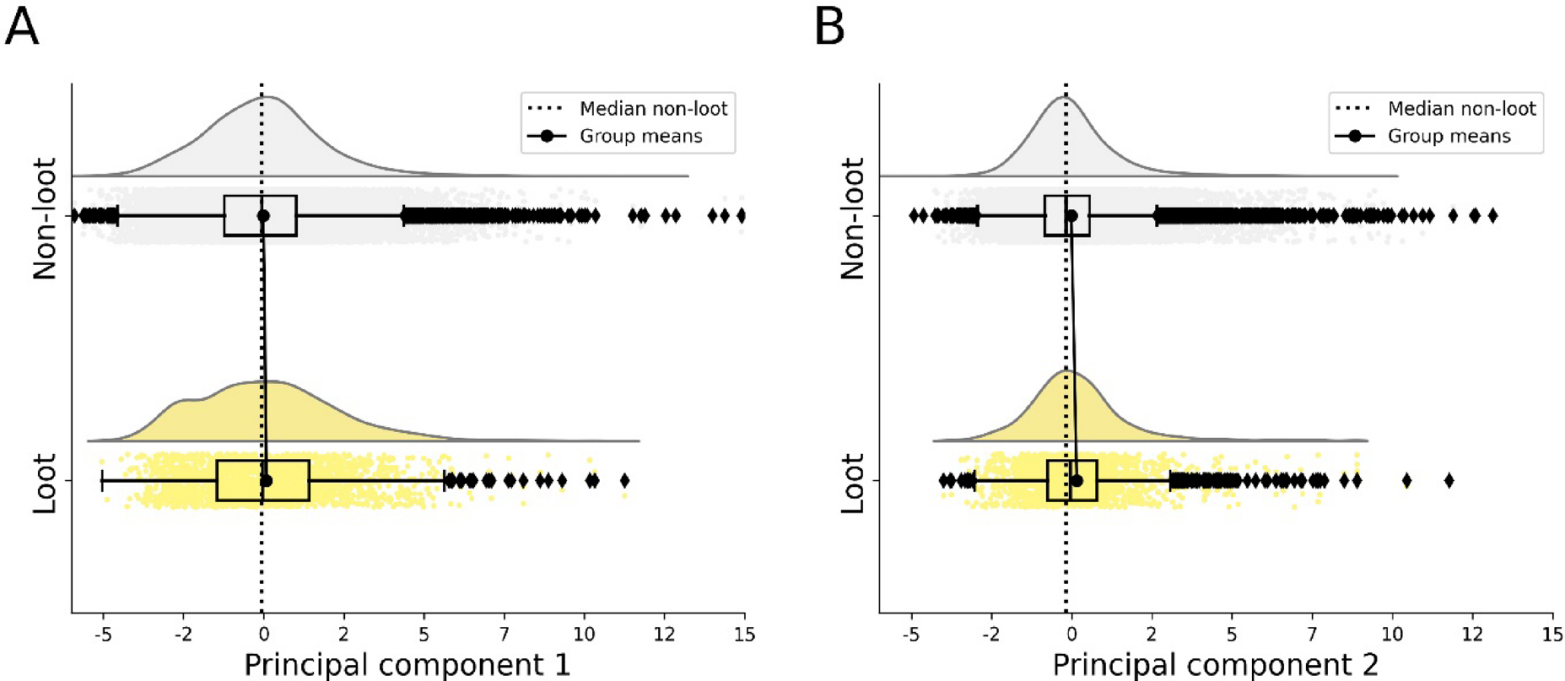
Distribution (cloud), scores (rain) and central tendency (box plot) of the eigenvalues for the first two principal components (panel **A** and **B**) for videos with and without loot boxes in grey and yellow, respectively. PC1 reflects overall and ongoing engagement, PC2 reflects relative engagement with videos.

A PCA was performed on the *z*-standardised data. Based on inspection of the scree plot (see Fig. S1 of the Supplementary Material), we considered the first two components for further analysis. The component values for loot and non-loot videos are listed in Table 3, the distributions are depicted in Fig. 3. Component loadings for all principal components are provided in Fig. 2. The first component (PC1) exhibited moderate to strong positive loadings for video views, likes and comments, and asymptote of the comment latency model. The component reflects, in absolute figures, how often a video is viewed, liked and commented on, and how long the video is interacted after its publication. We therefore term PC1 overall (active and passive) and ongoing engagement. The second component (PC2) was primarily constituted by like frequency and comment frequency, reflecting how many likes and comments a video received in relation its views. We therefore term PC2 relative engagement.

**Table 3:** Component values for the first principal components.

	Loot			Non-loot		
	*M*	*MD*	*SD*	*M*	*MD*	*SD*
PC1	0.0872	− 0.0281	2.1885	− 0.0084	− 0.0571	1.8860
PC2	− 0.0152	− 0.0399	1.5931	− 0.0152	− 0.1768	1.3926

The data were square-root transformed and within-channel z-standardised. PC1 reflects overall (active and passive) and ongoing engagement, PC2 reflects relative engagement.

*PC* principal component, *M* mean, *MD* median, *SD* standard deviation.

### Linear mixed models (LMMs)

Results from the LMMs revealed a positive and significant effect of loot box video content for the first principal component (PC1, *β* = 0.873, *SE* = 0.79, *p* = 0.008, see Table 4). This implies that, on average, videos featuring loot boxes exhibited higher overall and ongoing engagement than those without loot boxes, thus confirming the predicted engagement effect of loot box content. Notably, the size of the effect is rather small (see Fig. 3). However, looking at the raw video statistics, differences between loot and non-loot videos also manifest descriptively for all but two measures. For example, videos with loot box content had 54% more (absolute) views, 53% more (absolute) likes and 12% more likes per 1000 video views (like frequency), and 55% more comments per 1000 video views (comment frequency) compared to videos without loot box content (see Table 2). With regard to the second principal component, no significant effect of loot box content was observed (*β* = 0.167, *SE* = 0.256, *p* = 0.529, see Table 4).

**Table 4:** Linear mixed models (LMMs) for the first and second principal component.

	Estimate (β)	SD	z value	*p* value	Random effects		R^2^	
					Variance	SD	Marginal	Conditional
PC 1							0.016	0.038
Intercept	− 0.020	0.034	− 0.589	0.567	0.012	0.113		
Loot box video content	0.873	0.279	3.127	0.008*	0.970	0.985		
PC 2							0.001	0.036
Intercept	− 0.015	0.015	− 0.999	0.358	0.001	0.036		
Loot box video content	0.167	0.256	0.652	0.529	0.839	0.916		

The data were square-root transformed and within-channel *z*-standardised. PC1 reflects overall (active and passive) and ongoing engagement, PC2 reflects relative engagement.

*PC* principal component.

*p < 0.025, one-sided.

## Discussion

Building on the assumption that loot boxes resemble gambling, we tested the preregistered prediction that user engagement on YouTube gaming channels would be increased by loot box content in videos. To this end, we used web scraping to collect video statistics reflecting user engagement such as the number of views, likes and comments for videos posted in selected YouTube gaming channels. We condensed the measures using principal component analysis, and tested for differences in user engagement components for videos with and without loot box content, respectively.

As hypothesised, we found significant differences in component values for the first component, labelled as overall and ongoing engagement. For the second component, we found no evidence for differences in component values between videos with and without loot box content, respectively. The variables number of views, likes, comments and the asymptote of the comment latency model loaded most prominently on the first principal component. This component therefore reflects general quantitative user engagement, both passive (viewing), and active (commenting), as well as aspects of long-term interaction. A higher asymptote of the comment latency model for videos with compared to without loot box content indicates ongoing consumer engagement over longer time periods after a video is published.

For some games, players create videos of themselves opening large numbers of loot boxes (or card packs, depending on the game) shortly after a publisher has released new game content, e.g., an expansion pack. Some of this new content may have a significant impact on gameplay strategies, often altering or breaking the current most effective and dominant strategies in the game. Part of the enjoyment for viewers may come from watching whether the video creator acquires the most significant or impactful assets. Thus, interest in loot box opening should peak just after the release of new content, and should gradually decline as the expansion ages. Following this, the popularity and engagement with gameplay videos should be more consistent across the lifetime of an expansion when comparing it to the game’s overall interest timeline, which may include downtimes between expansions.

Not all forms of actions on social media reflect the same level of engagement. The COBRA framework differentiates between the three levels of consumption, contribution and creation, consumption reflecting minimal level of engagement (e.g. viewing), contribution reflecting medium level (e.g. liking, commenting), and creation highest level of engagement (e.g. publishing content)^21,22^. Like and comment counts load most strongly onto the first principal component, thus reflecting stronger medium-level engagement for videos with compared to without loot box content. Overall, the results suggest that passive, active and ongoing forms of user engagement are higher for videos with compared to without loot box content, confirming our hypothesis that loot box content is associated with elevated user engagement.

One possibility is that higher user engagement for loot compared to non-loot box content is linked to the gambling-like properties of loot boxes. Loot boxes are designed to keep players involved and investing money in a game and promote greater involvement in video games^20^. Thereby, potentially harmful content is incorporated into games in order to generate revenue. Critically, loot boxes are often found in games considered suitable for children, both in the mobile and PC/console games sector^2,11^. Our data show that greater involvement is not only found for games with loot boxes, but also when consuming gaming related video content on YouTube. We don’t know how old the users are, but we assume that many of them are teenagers^32^. This is a vulnerable phase for mental disorders, which is why loot content may be particularly problematic here.

The “gateway hypothesis” posits that video gaming and loot boxes may serve as a gateway activity into gambling and problem gambling^33–35^. While gaming and social media use may not problematic per se^36,37^, the exposure to videos featuring loot box openings may increase the frequency of gaming and/or gambling. In a longitudinal study of people aged 18–26, spending and risky loot box use predicted the start of gambling at a later point in time^9^. Our data do not provide any information on this, but this should be further investigated in future studies. Brooks and Clark’s^9^ analyses suggest that the relationship is driven by the exposure to randomised rewards rather than by performing microtransactions per se.

Some gamblers often report that not only gambling itself gives them pleasure, but watching other people gamble does also^38^. Receiving items from loot boxes in video games may trigger arousal^15^, and along similar lines, this may also apply to observing other people who receive items from loot boxes. Our analysis demonstrates that the gamblification of video games through loot boxes may drive increased user engagement with YouTube gaming videos. In light of the evidence for a positive relationship between engagement with loot boxes and problematic gaming and gambling^39^, the current analyses underline the need for recognising and analysing these links using also digital behaviour. While internet platforms such as YouTube collect massive amounts of user data to generate revenue from knowledge about correlations between content and user engagement with videos, these data contain information potentially relevant for research on problematic internet use and gambling behaviour. While the relationship between gambling-related content and social media engagement may be relatively straightforward as users engage with content directly related to gambling, using such data for research on problematic internet use requires more targeted approaches due to the broader nature of problematic internet use. Here, user-level data would be more informative in identifying problematic usage patterns. Still, social media activity may offer insights into problematic usage patterns on a broader scale by tracking excessive time spent on platforms or excessive engagement with specific content (e.g. by looking at posting frequency and timing patterns). Future research could benefit from combining platform-level data with user-level data.

### Limitations, strengths and perspectives

The current approach also has some limitations. First, it should be noted that the definition of views is not entirely transparent. YouTube does not disclose what duration of video viewing is counted as a view, or whether users are counted multiple times when watching a video repeatedly^40^. To reduce the influence of age of the videos, we also calculated relative user engagement measures. However, more likes will result in better findability of the video determined by the YouTube algorithm (which is not precisely known). Furthermore, more comments give more reasons for further comments. Therefore, ratios such as likes or comments per 1000 views are also subject to an influence of time.

The results of our work are to be viewed as specific for the games included in the analysis for several reasons. First, the selected games may have a more compelling display of loot boxes and reward structures than other games. Also, it may be the case that the gameplay videos of the games included are less compelling compared to loot box openings, or less compelling compared to games not included. Further, the ratio of games to YouTube content creators may have influenced the results. For some games, there may be a greater concentration in terms of the number of content creators that hold a certain percentage of viewership or hours viewed, while for other games, there may be a larger number of content creators that divide the fan base more evenly. This affects the popularity of certain games and content creators on YouTube, and in turn, the selection of games and channels for the current work. The results are therefore to be viewed as specific for the games included in the analysis and several prominent games could have been included (e.g. Genshin Impact and Clash Royale) but were simply overlooked during study preparation. Future research should explore a wider variety of games and genres.

A general shortcoming of the present approach (and similar web-scraping-based approaches in general) is that we cannot differentiate which people interact with the videos, and that the mechanisms underlying the difference in engagement for loot and non-loot content remain unclear. Coming from an observational study, the results do not allow any conclusions to be drawn about causality between loot box content and user engagement on the gaming channels. While increased engagement may reflect the gambling-like properties of loot boxes, it is also conceivable that higher engagement reflects enjoyment from watching other’s reacting to loot content. Loot box openings may generate tension and excitement and the mechanism driving higher engagement may, in part, resemble that of watching so-called reaction videos, for instance a desire to observe authentic reactions of others^41^. Nonetheless, regardless of the motivation to engage with loot box content, the present results highlight that loot box content is linked to higher engagement, which may drive exposure to and normalisation of gambling-like content in the context of gaming.

Data collection using web scraping offers many advantages for psychological research. It enables extracting large amounts of data from the internet in a comparatively short time, and data collection has no influence on the data itself. An advantage compared to survey-based research is the high ecological validity, since behaviour can be studied in a real environment. The current study demonstrates that such data contain information meaningful for behavioural addiction research. Data analysis should not only be left to the providers of these services, who adjust the content to maximise revenue, which may have negative effects for users. Finding higher user engagement for loot compared to non-loot box content may reflect the gambling-like properties of loot boxes. Building upon this, warning notices could be designed more effectively, since consumers perceive harm resulting from engaging with loot boxes lower than for many gambling activities^42^. Publicly available user data may serve as an early indicator of potential changes in problematic internet use and gambling behaviour. For instance, it would be valuable to identify individual factors associated with higher user engagement on gaming channels, since previous studies showed a particularly high increase in video game activities during the COVID-19 lockdown among male subjects^43^.

### Legal statement

The study was an observational study in which publicly available social media data were collected by means of web scraping. In Germany, web scraping for independent scientific purposes is generally legal, and no consent is required under the following conditions: The information to be evaluated must be generally accessible; technical measures designed to prevent web scraping may not be disregarded; the research may only serve non-commercial purposes; the use of web scraping technologies must not cause technical damage to the operator of the website^44,45^. The study’s approach aligned with these regulations. The application programming interface (API) and the respective key used for data collection granted access to publicly available data only. We did not disregard any technical measures designed to prevent web scraping and the procedure did not cause any damage to the website operator. Our research serves non-commercial purposes only. We did not collect data on individual user profiles and the data do not allow the identification of natural persons.

## Electronic supplementary material

Below is the link to the electronic supplementary material.

Supplementary Material 1

## Data Availability

The datasets generated and analysed during the current study are not publicly available, since in compliance with German laws, sharing data collected by means of web scraping is not permitted. Upon request, information on the data to reproduce the analyses will be shared from the corresponding author on reasonable request. The code for analysis is openly available at https://osf.io/bjndc/.
