# Supplementary material for: Increased user engagement on YouTube for loot box content and its potential relevance for behavioural addictions
Source: Sci Rep. 2025 May 15;15:16833. doi: 10.1038/s41598-025-01482-5 (PMC12078526; doi:10.1038/s41598-025-01482-5)
Supplement: Supplementary file 1 — Supplementary Material 1 [file 41598_2025_1482_MOESM1_ESM.pdf]

Supplement

Increased user engagement on YouTube for loot box content and its potential relevance for behavioural addictions

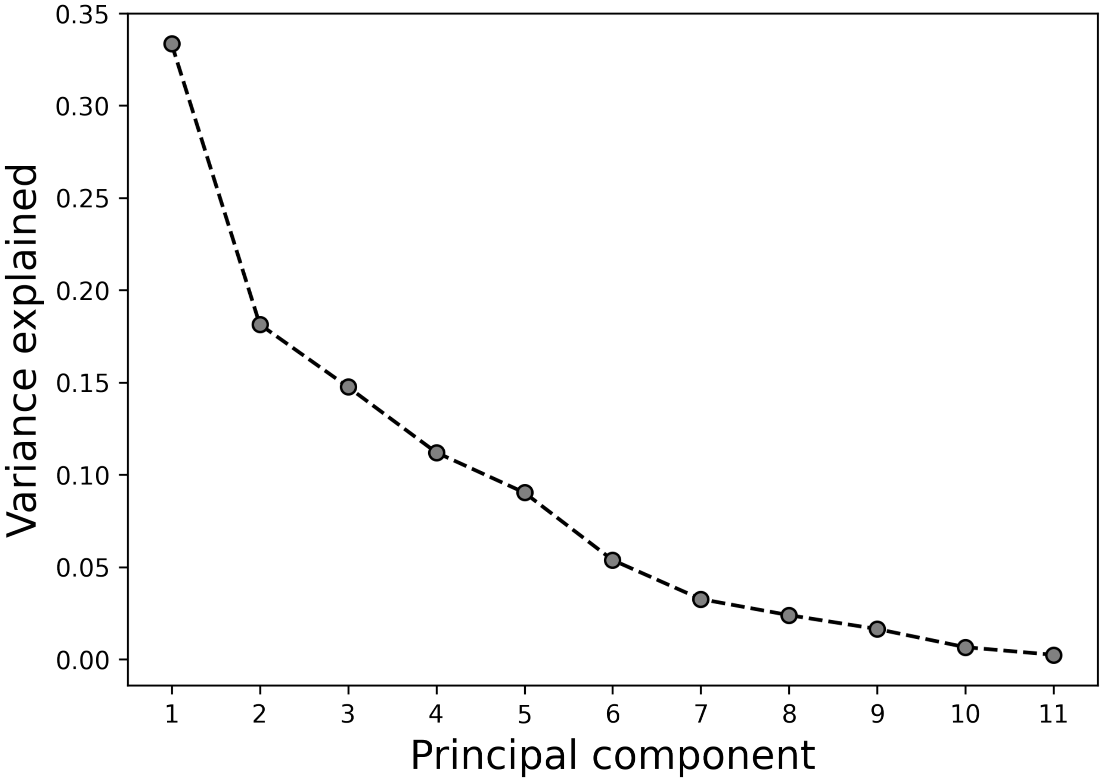

Figure S1: Screeplot depicting the variance of the data set explained by the principal components.

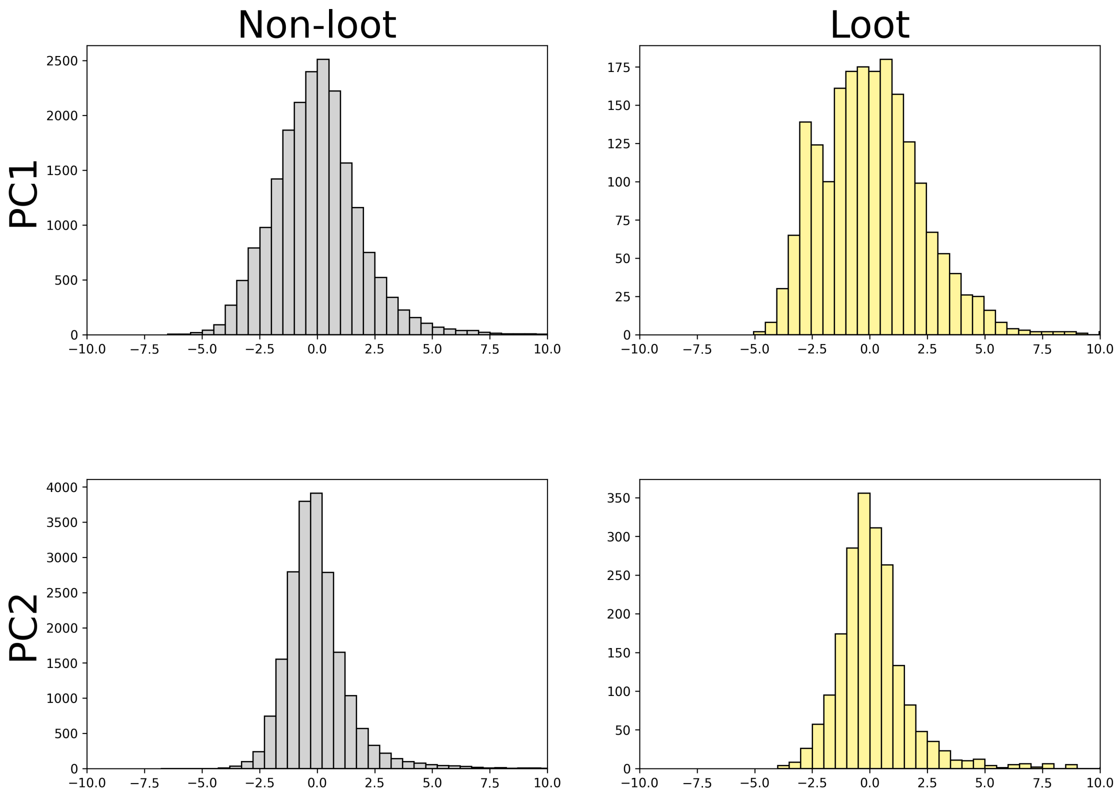

Figure S2: Distribution plots of the first two principal components (square-root transformed data) for videos without and videos with loot box content in grey and yellow, respectively.

Supplement

Increased user engagement on YouTube for loot box content and its potential relevance for behavioural addictions

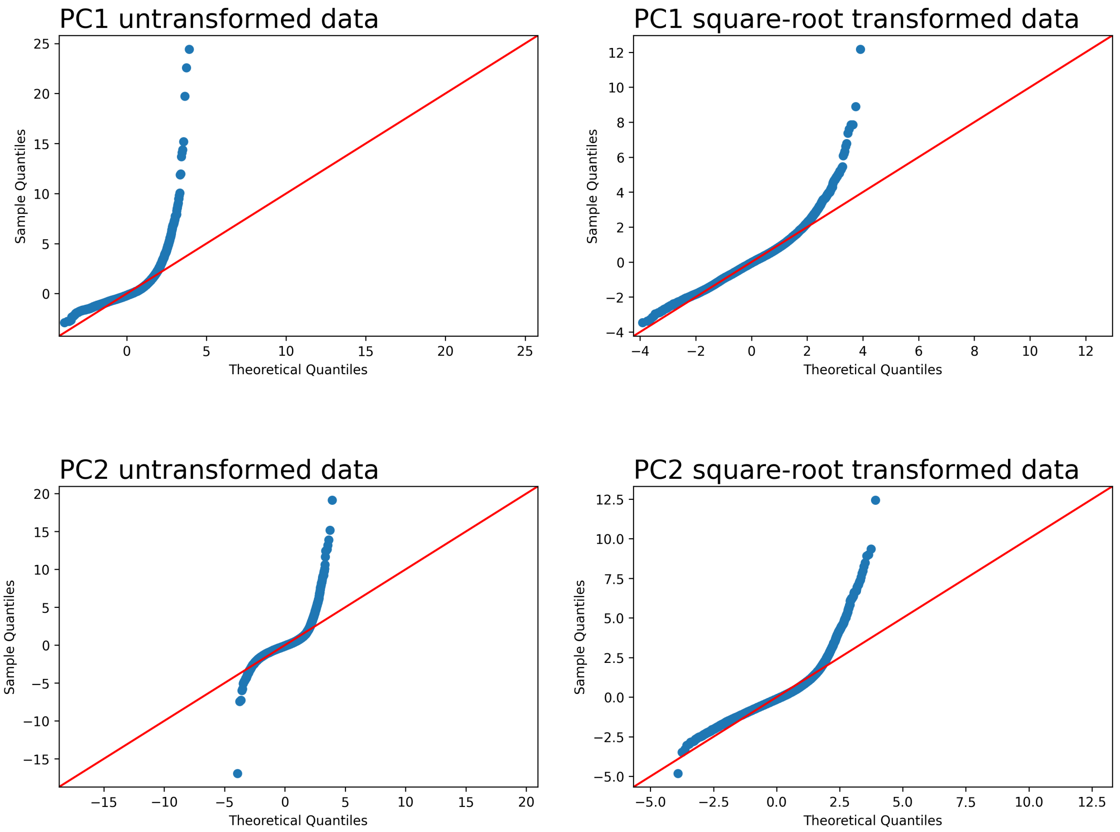

Figure S3: Quantile-qantile plot for the principle components before (left column) and after (right column) square-root transformation.
